# Effects of TCM on polycystic ovary syndrome and its cellular endocrine mechanism

**DOI:** 10.3389/fendo.2023.956772

**Published:** 2023-05-16

**Authors:** Huize Chen, Chujun Deng, Zeyu Meng, Shengxi Meng

**Affiliations:** ^1^ Department of Traditional Chinese Medicine, Shanghai Sixth People’s Hospital Affiliated to Shanghai Jiao Tong University School of Medicine, Shanghai, China; ^2^ The Second Clinical Medical College, Heilongjiang University of Chinese Medicine, Harbin, China

**Keywords:** polycystic ovary syndrome, TCM, cellular endocrinology, mechanism of action, metabolic disorders

## Abstract

Polycystic ovary syndrome (PCOS) is a reproductive endocrine disease characterized by menstrual disorders, infertility, and obesity, often accompanied by insulin resistance and metabolic disorders. The pathogenesis of PCOS is relatively complex and has a certain relationship with endocrine disorders. The increase of androgen and luteinizing hormone (LH) is the main cause of a series of symptoms. Traditional Chinese medicine (TCM) has obvious advantages and significant curative effects in the treatment of this disease. It can effectively reduce the insulin level of PCOS patients, regulate lipid metabolism, and increase ovulation rate and pregnancy rate and has fewer side effects. This article reviews the efficacy and safety of Chinese herbs and other TCM (such as acupuncture) in the treatment of PCOS and its complications in recent years, as well as the effect and mechanism on cellular endocrine, in order to provide a new clinical idea for the treatment of PCOS.

## 1 Introduction

Polycystic ovary syndrome (PCOS) is one of the common endocrine diseases in women of childbearing age, which seriously affects their reproduction, metabolism, and psychology. The clinical manifestations of PCOS are mainly irregular menstruation, hyperandrogenemia (HA), ovulation dysfunction infertility, polycystic ovary, and hormonal changes, which affect patients’ fertility, quality of life, and long-term health. The global prevalence is estimated to range from 4% to 12% due to poor eating habits and unprecedented psychological and social stress (1). In addition, a large number of women with PCOS symptoms are not clearly diagnosed. The prevalence of diabetes in the PCOS population is approximately 26.5%, which significantly reduces the quality of life of patients and imposes a high cost on the healthcare system. It has been reported that the medical-related economic burden of PCOS in the United States is $4.36 billion per year (2). Different pharmaceutical treatments have been proposed for PCOS. However, they also have drawbacks, such as adverse reactions, low patient compliance with long-term medication, low efficacy, and contraindications in some cases (3, 4). Traditional Chinese medicine (TCM), as a complementary and alternative treatment method, is an effective way.

PCOS is an endocrine and metabolic disorder caused by the interaction of heredity and the environment. Its etiology and pathogenesis have not been elucidated completely yet (5). Previous studies have suggested PCOS may be linked to mood and mental disorders (6). Emotional factors can affect the changes of the hypothalamic–pituitary–ovarian axis (HPOA) and cause neuroendocrine changes, resulting in irregular ovarian ovulation and PCOS. Some studies have suggested that the occurrence of PCOS is related to DNA methylation, X chromosome inactivation, and histone modification (7). Some scholars have found that the anti-Müllerian hormone (AMH) is associated with the etiology of PCOS, and the concentration of AMH can predict the ovarian response in ovulation induction (8). In recent years, alterations in intestinal flora have been found to be involved in the pathogenesis of PCOS. It can lead to insulin resistance (IR) and influence androgen metabolism and follicle development (9). Inflammation is also one of the risk factors, and any disturbance of the level of inflammatory factors may lead to the dysfunction of ovarian function (10). In addition, a meta-analysis suggested a significant correlation between PCOS and prostate-specific antigen (PSA), and the role of PSA in patients with PCOS cannot be ignored clinically (11).

There is no disease name of PCOS in ancient Chinese medicine books. According to its clinical manifestations, it can be classified as “irregular menstruation”, “amenorrhea”, or “infertility” category. The treatment mainly focuses on restoring the balance between the kidney, Tiangui, Chongren, and uterus. Tiangui is a sexual stimulant for women, which is essential for women’s menstruation and pregnancy, and its function is similar to neuroendocrine hormones that regulate reproduction. The disturbance of the time, state, and rhythm of Tiangui can lead to female reproductive problems, and ovarian dysfunction in PCOS patients can show Tiangui disorders. TCM has a good curative effect and high safety in the treatment of PCOS, which has attracted more and more attention (12). However, the biological mechanism has not been clearly elucidated, and the treatment protocols are still controversial. This paper reviewed the effects of traditional Chinese as a treatment for PCOS and introduced its potential cellular endocrine mechanism in order to provide a reference for the clinical treatment and research of PCOS.

## 2 Treatment of PCOS in TCM

### 2.1 Treatment of PCOS by acupuncture

Acupuncture is an indispensable part of traditional medicine and has been used in China for more than 3,000 years. Acupuncture has clinical efficacy in the treatment of cardiovascular diseases, epilepsy, anxiety, circadian rhythm disorders, PCOS, low reproductive capacity, and autonomic nervous system diseases (13). With the modernization of TCM and its advantages, such as convenient operation and economic and satisfactory results, more and more countries are beginning to apply it to treat and prevent diseases. Acupuncture was also not confined to its original model and gradually evolved into electroacupuncture (EA), warm needle, acupoint insertion, and ear point, and they have been used in the treatment of PCOS (14, 15). Currently, a large number of clinical and animal trials have shown that acupuncture can regulate the function of the HPOA and metabolism in PCOS, promote ovulation, and improve IR and endometrial receptivity (ER) (16).

Acupuncture is a TCM therapy in which very thin metal needles are inserted into specific parts of the body. Acupuncture can control the function of the autonomic nervous system (ANS); it can balance the sympathetic and parasympathetic nerve activity, regulate the adaptive neurotransmitters in related brain regions, and reduce the autonomic response, which has been proved to have a certain curative effect on PCOS patients (17, 18). Professor Shi treated PCOS women with obesity and infertility by acupuncture from the perspective of spleen and kidney function. The pathogenesis was a deficiency of the spleen and kidney as the primary and obstruction of phlegm-dampness and collaterals as the secondary. According to clinical syndrome differentiation, the main acupoints are selected from the meridian, pregnancy meridian, kidney meridian, and spleen and stomach meridian, and the corresponding acupoint prescriptions and techniques are used. The purpose is to tonify the kidney, strengthen the spleen, nourish qi, dissolve phlegm, remove dampness and remove stasis, promote blood circulation to restore menstrual flow, and help pregnancy (19). Professor Shi put forward the theory of “treatment based on syndrome differentiation, equal attention to the nature of acupoints and medicines, similar acupoints and prescriptions, and determination of reinforcing and reducing methods”. It fully embodies the idea of TCM “treatment based on syndrome differentiation” and shows the curative effect of acupoints and acupuncture techniques (19). EA can inhibit the overexpression of AMH and increase the expression level of P450arom in ovarian granulosa cells (GCs), thereby re-establishing the dependence of follicular development on follicle-stimulating hormone (FSH) and improving follicular dysplasia and hyperandrogenemia in PCOS patients with kidney deficiency and phlegm-dampness (20). In addition, acupuncture can improve vascular endothelial function, regulate dyslipidemia, correct IR, and improve endocrine disorders (21). EA has similar effects to metformin (22) and alleviates anxiety and depression in PCOS patients by regulating serum β-endorphin and androgen levels (23).

The acupuncture method of “regulating pregnancy and du-pulse” can improve the menstrual cycle, increase endometrial thickness, promote oocyte growth and follicle development, reduce serum luteinizing hormone (LH) level, improve ovarian function, and increase ovulation rate in PCOS patients (24, 25). Acupuncture can affect the production of β-endorphin, gonadotropin-releasing hormones, ovulation, and the menstrual cycle (26). Cao Y et al. conducted a randomized controlled trial (RCT) on 60 PCOS patients and found that LH/FSH ratio, LH, and total testosterone (TT) were significantly reduced after 12 weeks of acupuncture treatment, as well as significant improvements in body mass index (BMI), menstrual times, and polycystic ovary number (27). Huang S et al. found that personalized acupuncture can improve live birth rates (LBRs) in infertile women with PCOS in a multicenter RCT (28). However, in 2017 and 2018, the *Journal of American Medical Association* published two trial reports examining the potential effect of acupuncture on improving *in vitro* fertilization (IVF) or LBR in women with PCOS. The study found that acupuncture did not increase LBR, which may be due to unexpected biases in the study, such as using ineffective control interventions and underestimating the effect of real acupuncture (29). A 1,000-sample RCT trial also showed that acupuncture did not increase the rate of live births in infertile patients (30).

The standard treatment of PCOS includes oral medications, lifestyle changes, and surgery. Pharmacology-based treatments are effective in only 60% of patients. Therefore, acupuncture provides an alternative (14). In women with PCOS and IR, acupuncture was superior to metformin in improving glucose metabolism and had a lower incidence of gastrointestinal adverse effects (31). On the basis of Western medicine treatment of PCOS, adding acupuncture treatment can improve the curative effect and shorten the course of the disease. Letrozole combined with EA and TCM can significantly improve the menstrual cycle and reduce body weight, LH, LH/FSH, testosterone (T), and AMH levels. The therapeutic effect is superior to that of Western medicine alone, and there are no adverse reactions (32), based on the research of Jo J et al. (33). Acupuncture combined with TCM could improve the endocrine level and IR of phlegm-dampness PCOS and reduce miR-29 expression and TCM symptom score (34).

Modern acupuncture treatment of PCOS mainly includes Sanyinjiao (SP 6), Guanyuan (CV 4), Zigong (EX-CA 1), Zhongji (CV 3), and Qihai (CV 6). Points were selected based on the theory of meridians and zang-fu organs, syndrome differentiation, and meridian circulation. In addition, the method of selecting adjacent points was also adopted. The main acupoints are located in the fetal pulse, the spleen meridian of Foot Taiyin, and the stomach meridian of Foot Yangming. In the special acupoints, Zheng Mu, Wu Shu, and Bei Shu are used more. Generally, five to seven acupuncture points are taken (35). Although the mechanism of acupuncture treatment for PCOS has been found in modern medicine, most of the trials have small sample sizes and lack consistency in the selection of acupoints. Larger, long-term randomized controlled trials are needed in the future to provide standardized protocols. A summary of the effect of acupuncture on PCOS outcomes is shown in **Table 1**.

**Table 1:** Summary of a randomized study on the effect of acupuncture on PCOS outcomes.

Intervention	Sample size	Group	Duration of study	Results	Year, reference
EA	62	Intervention group: EA + moxibustion Control group: moxibustion	3 months	Inhibit the overexpression of AMH, increase the expression level of P450arom in ovarian granulosa cells, improve abnormal follicular development and hyperandrogenemia	2021 (20)
Acupuncture	140	Impaired glucose tolerance (IGT) group: acupuncture Normal glucose tolerance (NGT) group: acupuncture	3 months	The TCM symptom score, asymmetric dimethylarginine (ADMD), endothelin-1 (ET-1), malondialdehyde (MDA), FPG, 2hPG, FINS, and HOMA-IR of the patients decreased, while NO increased	2021 (21)
EA	70	EA group: EA Medication group: metformin	3 months	Serum T, HOMA-IR, LDL, TG, and TC levels were significantly decreased, while HDL level was increased. The effect of acupuncture was better than that of metformin	2020 (22)
Acupuncture	40	Observation Group: acupuncture + lifestyle interventions Control group: lifestyle interventions	4 months	BMI, ferriman-gallway (F-G) score, self-rating anxiety scale (SAS) score, self-rating depression scale (SDS) score, and serum free androgen index (FAI) level were decreased (FAI), while free androgen index (PCOSQ) score, serum SHBG, and β-endorphin levels were increased. Acupuncture treatment of PCOS patients can effectively relieve anxiety and depression	2020 (23)
Acupuncture	60	Acupuncture group, acupuncture CPA/EE group: ethinylestradiol	3 months	The LH/FSH ratio, LH, and TT were significantly decreased, and BMI, menstrual frequency, and polycystic ovary number were significantly improved	2019 (27)
EA	120	Group A: Diane-35 Group B: CHF + Diane-35 Group C: CHF + Diane-35 + EA	3 months	The levels of LH, LH/FSH, T, and AMH decreased, and the ovulation and pregnancy rates increased	2018 (32)
Acupuncture	60	Observation group: letrozole and metformin Control group: letrozole and metformin + CHF + acupuncture	Three menstrual cycles	Insulin resistance and pregnancy rate were improved, and Mir-29 expression and TCM symptom score were decreased	2017 (34)
Acupoint paste	80	Research group: CHF + acupoint paste Control group: CHF	/	Improve obesity and endocrine function	2021 (36)
Acupoint catgut embedding	62	Group 1: CHF Group 2: acupoint catgut embedding Group 3: acupoint catgut embedding + CHF	3 months	The levels of fast insulin, HOMA-IR, TG, and TC were significantly decreased, while the HDL level, cyclic ovulation rate, and clinical pregnancy rate were higher	2016 (37)

PCOS, polycystic ovary syndrome; EA, electroacupuncture; TCM, traditional Chinese medicine; FPG, fasting plasma glucose; 2hPG, 2-h postprandial blood glucose; HOMA-IR, Homeostatic Model Assessment for Insulin Resistance; LDL, low-density lipoprotein; TG, triglyceride; TC, total cholesterol; HDL, high-density lipoprotein; BMI, body mass index; SHBG, sex hormone-binding globulin; FSH, follicle-stimulating hormone; LH, luteinizing hormone; TT, total testosterone; CHF, Chinese herbal formulae.

### 2.2 Treatment of PCOS by Chinese herbal formulae

Due to the relatively complex pathogenesis of PCOS, there is no single drug that can control all symptoms. Existing pharmaceutical formulations, such as oral contraceptives (OCs), have been suggested as first-line treatments for menstrual irregularities. However, OCs are not appropriate for pregnant women. Insulin sensitizers can reduce insulin levels and hyperandrogenemia in PCOS patients, but the incidence of gastrointestinal adverse reactions is higher. In China, it is a common practice for TCM to treat gynecological problems and infertility. Current studies have shown that Chinese medicine has a beneficial effect on the treatment of PCOS. The database analysis of the Taiwan National Health Insurance Plan showed that 89.22% of women who were newly diagnosed with PCOS had received TCM treatment (38). Jiawei Xiaoyao Powder and Xiangfu are the most commonly used compound and single medicinal materials, respectively (38). Several studies have shown that TCM has similar safety and clinical effects in the treatment of PCOS (39, 40).

According to the main pathogenesis of PCOS, TCM treatment should focus on tonifying the kidney and simultaneously treating the liver, spleen, and heart. Professor Shi Yin summarized the clinical experience and proposed the method of “staging, classification and sorting” for PCOS treatment. The stage should take the law of follicle development as the core and follow the rule of Yin and Yang. In terms of classification, emphasis is placed on individual treatment according to obesity, wasting, non-obesity, and fertility. In addition, psychological counseling and life adjustment are very important for patients, as the unity of body and mind can improve the curative effect (41).

Kidney nourishment and phlegm removal (KNPR) effectively improved the glucose and lipid metabolism of obese PCOS rats. The levels of fasting blood glucose (FBG), total cholesterol (TC), triglyceride (TG), low-density lipoprotein (LDL), and free fatty acid (FFA) in serum were decreased, and the expression of high-density lipoprotein (HDL) was increased (42). Chen WJ et al. found that Quyu Huatan Decoction has good clinical efficacy in treating PCOS. It can not only improve the secretion of various sex hormones but also regulate the body’s sugar and lipid metabolism (43). In addition, this method can improve hyperandrogenemia, the number of granulosa cell layers and luteal tissue, and regulate LH/FSH ratio (44). Zhibai Dihuang Decoction (MZBDD) has a dose–effect relationship in the treatment of PCOS hyperandrogenism (45). Pan X et al. found that Bushen Jieyu Tiaochong Formula (BJTF) could improve abnormal follicular dilation in PCOS rats and reduce the levels of free testosterone (FT), LH, and LH/FSH ratio in serum. BJTF can relieve chronic psychological stress behavior and regulate brain monoamine neurotransmitter expression and metabolism. The apoptosis index of GCs, glucose-regulated protein 78 (GRP78), CHOP, and ATF4 was decreased (46).

Yu J et al. showed that Yushi Qinggan Recipe (YQR) could improve the clinical symptoms of PCOS patients, regulate their endocrine levels, and promote ovulation and pregnancy. The levels of LH, LH/FSH, T, FT, dehydroepiandrosterone sulfate (DHEAS), insulin (INS), and insulin area under the curve (IAUC) were all significantly reduced. At the same time, the symptoms of acne, irregular menstruation, irritability, breast distension, dry mouth and bitter mouth, greasy hair/alopecia, and constipation were improved (47). Bushen Huoxue Culuan Prescription can reduce LH level and increase FSH level, improve polycystic ovary, increase the number of follicles, facilitate the development of mature follicles, and improve ovulation rate (48). In addition, TCM can downregulate reactive oxygen species (ROS) protein expression in GCs, correct oxidative stress *in vivo*, and improve the rate of high-quality embryos (49). Bushen Tiaojing decoction combined with Western medicine can improve endometrial thickness and shape, promote menstrual recovery, induce ovulation, and enhance endometrial permeability. The levels of T, LH, Homeostatic Model Assessment for Insulin Resistance (HOMA-IR), and leptin (LP) in patients were reduced, and the efficacy was better than in Western medicine alone (50). Bushen Tiaochong Decoction has good efficacy in the treatment of obesity PCOS, which can significantly improve the level of a reproductive endocrine hormone, reduce BMI, and improve endometrial acceptance. It is a safe and promising treatment method (51).

TCM can regulate endocrine and improve menstrual irregularity, ovulation, and pregnancy rate by regulating ovarian hemodynamics, serum hormone levels, and menstruation in PCOS patients (52, 53). TCM’s clinical curative effect is better than that of Western medicine treatment (54–56). A meta-analysis showed that the efficacy of TCM combined with letrozole (LE) in the treatment of PCOS was superior to LE alone in regulating ovulation rate, pregnancy rate, number of mature follicles, endometrial thickness, cervical mucus score, serum FSH, LH, E2, T, and prolactin (PRL) levels (57, 58). On the basis of the control group, Bailingtiaogan decoction can obviously improve the clinical symptoms, increase the endometrial thickness, reduce ovarian volume, and improve the clinical pregnancy rate. FFA and C-reactive protein (CRP) levels were significantly lower than those of the control group; the difference was statistically significant (p < 0.05) (59). Wei A et al. showed that the Dingkun pill combined with clomiphene in the treatment of PCOS with infertility is more effective than clomiphene alone (60). Although the addition of Chinese herbal formulae (CHF) to clomiphene may improve pregnancy rates, there are no relevant data to support live birth rates in subfertile women with PCOS. In addition, the evidence on adverse effects is insufficient to indicate whether CHF is safe. In the future, well-designed, well-conducted randomized controlled trials will be needed, with a special focus on live birth rates and other safety indicators (61). TCM cycle therapy combined with Dian-35 treatment has fewer adverse reactions (62). CHF combined with metformin hydrochloride was superior to Western medicine alone in improving the total effective rate of blood glucose and hormone of PCOS. After treatment, serum FSH, LH, LH/FSH, and T changed significantly. FBG, 2-h postprandial blood glucose (2hPG), and BMI were decreased, and the changes in the treatment group were more significant than those in the control group (p < 0.05) (63). No adverse effects of CHF on liver and kidney function were observed in PCOS patients (64).

More and more studies have proved that IR regulates multiple mediators and pathways and is seriously involved in the pathogenesis and development of PCOS (65, 66). A study by Liang RN et al. showed that Heyan Kuntai Capsule (HYKT) can improve glucose and lipid metabolism disorder, IR, and insulin sensitivity in PCOS patients, and its effect is similar to that of insulin sensitizer. After HYKT treatment, BMI and waist–hip ratio (WHR) decreased. Fasting plasma glucose (FPG), 2hPG, and serum sex hormones, such as LH, LH/FSH, and T, were decreased. TC, TG, LDL, INS, and HOMA-IR in the HYKT group were significantly lower than those in the placebo group, while HDL and insulin sensitivity index (ISI) were higher (67). CHF can improve glycolipid metabolism in non-obese PCOS patients by reducing LH levels and LH/FSH (68). Qiu Z et al. found that after Liuwei Dihuang Pills (LWDH Pills) treatment, ISI returned to normal; serum levels of FSH, E2, and progesterone (P) were significantly increased; and LH and T levels were decreased. Polycystic ovarian changes and follicular atresia were reduced (69). Heqi San also has a similar effect (70). In PCOS patients, Dingkun Pill (DKP) or DKP in combination with Diane-35 resulted in a slight improvement in insulin sensitivity (71). Heat-clearing drugs can obviously improve IR and reduce serum LH, T, and PRL levels and TCM syndrome score, and their efficacy is better than that of metformin. Compared with before treatment, BMI, fasting insulin (FINS), 2-h INS, HOMA-IR, LP, LH, PRL, T, and TCM syndrome scores were significantly decreased, while adiponectin (APN) level was increased, and the differences were statistically significant (p < 0.05) (72). The imbalance of intestinal flora is correlated with the incidence of PCOS. The number of butyric-producing bacteria decreased, and the number of lipopolysaccharide-producing and pro-inflammatory bacteria increased in PCOS patients. Jiawei Qigong pill can increase the diversity of intestinal flora and the number of probiotics and improve the structure of intestinal flora and IR (73).

Oxidative stress and inflammation are related to the occurrence of PCOS (74). Lu C et al. confirmed that Bushen Huatan Formula (BHF) can reduce the inflammatory response and oxidative stress of PCOS. After BHF intervention for three menstrual cycles, serum glycerophosphorylethanolamin (GPEA), creatinine, and creatinine levels were decreased in the normal insulin group (NI) and hyperinsulin group (HI). The changes in phospholipid metabolism were mainly observed in the NI group. In the HI group, lysine, phenol sulfate, and phenylpropofol decreased, while ornithine, proline, and acetylcholine increased (75). Cangfu Daotan Decoction (CFDTT) can regulate lipid metabolism, sex hormone secretion, and inflammatory response; CFDTT intervention reduced serum TC, TG, LDL-C, LH, T, IL-1β, IL-6, and TNF-α levels. The levels of HDL-C, FSH, and E2 were increased in a dose-dependent manner. CFDTT induced the expression of organic anion transporting polypeptides (OATPs) in ovarian and uterine tissues (76). TCM can downregulate the expression of apoptosis-related proteins to repair ovarian lesions and improve cell apoptosis (77). Cangfudaotan decoction (CFD) treatment can improve IR, restore serum hormone levels, inhibit inflammatory cytokines, and reduce ovarian morphological damage in PCOS rats. In GCs of PCOS, the cell viability was improved, and apoptosis was inhibited after CFD treatment (78). Chronic stress induces endocrine disorders, leading to the development of PCOS. Xiao-yao-san (XYS) treatment ameliorated the irregular estrus cycle and abnormal follicular development induced by chronic unpredictable mild stress (CUMS). E2, P, and LH levels were decreased, and granulosa cell apoptosis and autophagy were inhibited. β-Hydroxylase, c-Fos, and norepinephrine (NE) were reduced. XYS can be used as a potential therapeutic strategy, and its beneficial effects are related to the regulation of sympathetic nerve activity (79). A summary of the effect of CHF on PCOS outcomes is shown in **Table 2**.

**Table 2:** Summary of a randomized study on the effect of CHF on PCOS outcomes.

Intervention	Sample size	Group	Duration of study	Results	Reference
Herbal medicine (*Cinnamomum verum*, *Glycyrrhiza glabra*, *Hypericum perforatum*, and *Paeonia lactiflora*/*Tribulus terrestris*)	122	Intervention group: CHF Control group: lifestyle modification	3 months	Body mass index, hormone levels, depression, anxiety, and pregnancy rates all improved	2017 (40)
Quyu Huatan decoction	93	Control group: metformin Observation group: CHF	/	Improve the secretion of all kinds of sex hormones but also regulate the body’s sugar lipid metabolism	2016 (43)
Modified Zhibaidihuang decoction	120	Low-dosage group: CHF Medium-dosage group: CHF High-dosage group: CHF Control group:/	4 weeks	There is a dose–response relationship in the treatment of PCOS androgen hyperplasia	2018 (45)
Yushi Qinggan Recipe	175	Treatment group: CHF Control group: Diane-35	3 months	The levels of LH, LH/FSH, T, FT, DHEAS, INS, and IAUC were significantly reduced, effectively promoting ovulation and pregnancy	2019 (47)
Bushen Huoxue Culuan Prescription	80	Treatment group: CHF Control group: clomiphene	3 to 6 months	Reduce LH level and increase FSH level, improve polycystic ovary, increase the number of follicles, improve ovulation rate	2017 (48)
Bushen Tiaojing decoction	114	Observation group: ethinylestradiol + CHF Control group: ethinylestradiol	3 menstrual cycles	Improve endometrial thickness and shape, promote menstrual recovery, induce ovulation, enhance endometrial permeability	2015 (50)
Bushen Tiaochong decoction	147	Treatment group: cyproterone + CHF Control group: cyproterone	3 menstrual cycles	The levels of LH, FSH, and LH/FSH decreased significantly, and E2 increased, which increased the level of a reproductive endocrine hormone, reduced BMI, and improved endometrial acceptance	2018 (51)
Menshi Xiaonang Yin; Tanshinone Capsules	60	TCM group: Menshi Xiaonang Yin comprehensive group: Menshi Xiaonang Yin + Tanshinone Capsules	6 months	Decrease serum T level and acne score, increase ovulation rate and pregnancy rate	2018 (52)
Yangyinshugan capsule	100	Observation group: clomifene citrate + CHF control group: clomifene citrate	3 menstrual cycles	The ovarian hemodynamics and serum hormone levels were improved, and the pregnancy rate was increased	2018 (53)
Dihuang Wan combined with Xionggui Erchen Tang	120	Observation group: CHF Control group: ethinylestradiol	3 menstrual cycles	Regulate endocrine, promote menstrual recovery and spontaneous ovulation	2018 (54)
Bailingtiaogan decoction	110	Observation group: ethinylestradiol + CHF Control group: ethinylestradiol	Four menstrual cycles	Improve clinical symptoms, increase endometrial thickness, reduce ovarian volume, improve clinical pregnancy rate	2016 (59)
Xiaozhi Decoction	60	Treatment group: metformin + CHF Control group: metformin	3 months	There were significant changes in serum levels of FSH, LH, LH/FSH, and T, as well as decreases in blood glucose and BMI	2017 (63)
Modified Zhibai Dihuang Decoction	45	MZBDHD group: CHF DG group: Diane-35	/	Promote natural ovulation	2018 (64)
Heyan Kuntai Capsule	100	HYKT group: CHF Placebo groups: placebo	6 months	It can improve glucose and lipid metabolism disorder and insulin resistance in PCOS patients and improve insulin sensitivity	2019 (67)
Bushen Tiaogan Recipe	60	Treatment group: CHF Control group: Metformin	3 months	Decrease LH level and LH/FSH and improve glucose and lipid metabolism in non-obese PCOS patients	2017 (68)
Dingkun Pill	117	Group A: CHF B: Diane-35 Group C: Diane-35 + CHF	3 months	Improved insulin sensitivity	2019 (71)
Bushen Huatan Formula	30	Normoinsulinemic group: CHF Hyperinsulinemic group: CHF	Three menstrual cycles	GPEA and creatinine levels decreased and phospholipid metabolism changed	2016 (75)

CHF, Chinese herbal formulae; PCOS, polycystic ovary syndrome; LH, luteinizing hormone; FSH, follicle-stimulating hormone; FT, free testosterone; DHEAS, dehydroepiandrosterone sulfate; IAUC, insulin area under the curve.

### 2.3 Effects of a single herb or herbal extract on PCOS

In an open-label, one-arm, non-randomized, post-marketing surveillance study, *Trigonella foenum-graecum* seed extract caused ovarian cysts reduction or dissolution in PCOS patients, 71% of patients returned to normal menstrual cycles after treatment, and 12% became pregnant subsequently (80). Cinnamon can improve the menstrual cycle and ovarian size of patients and significantly reduce serum FPG, INS, HOMA-IR, TC, and LDL levels. Short-term cinnamon supplementation had beneficial effects on metabolism; it may be an effective treatment option for some PCOS women (81–84). Miao M et al. found that Dodder Total Flavone can reduce the expression of androgen receptor (AR) in HPOA of PCOS rats. Compared with the PCOS model rat, the levels of serum T, GnRH, and LH; ovarian index; and LH/FSH ratio were decreased in Dodder Total Flavone high-dose, medium-dose, and low-dose groups, while the levels of E2 and FSH were increased. To some degree, the pathological changes of cortical thickening, collagen, increased follicular atresia, and lutein in ovarian tissues were alleviated (85).

Berberine is a quaternary ammonium salt from the protoberberine group of isoquinoline alkaloids. It is found in plants such as *Hydrastis canadensis*, *Berberis vulgaris*, *Coptis chinensis*, *Xanthorhiza simplicissima*, and *Eschscholzia californica*. It has obvious anti-inflammatory and antioxidant effects *in vitro*. In PCOS animals, berberine has neuroprotective and cardiovascular effects; the effects of lowering lipids and improving IR have been clearly demonstrated in the RCT. In addition, preliminary clinical evidence suggests that berberine can reduce endothelial cell inflammation and improve vascular health (86). Berberine has the same effect as metformin in alleviating IR and improving glucolipid metabolism and reproductive endocrine status (87), and it is also beneficial to the reproductive rate of PCOS infertile patients (88). *C. chinensis* has the function of clearing heat and detoxifying, reversing pathological damage of PCOS ovarian tissue, and regulating the expression of inflammatory factors (89). Quercetin can reduce the levels of INS, IL-1β, IL-6, TNF-α, and NF-κB nuclear translocation. It has a good therapeutic effect on PCOS rats (90). Curcumin decreased BMI, FPG, INS, and IR significantly. In addition, taking curcumin can upregulate peroxisome proliferator-activated receptor gamma (PPAR-gamma) gene, PGC1 α gene, low-density lipoprotein receptor (LDLR) gene expression, and Gpx enzyme activity. Moreover, curcumin can effectively reduce oxidative stress-related complications and increased insulin sensitivity in PCOS patients (92–94).

Chinese herbal medicine has certain benefits in improving IR. *Salvia officinalis* extract can reduce the BMI and systolic blood pressure of PCOS patients and improve insulin resistance indicators (95). Soy isoflavones for 12 weeks in PCOS women significantly improved IR, hormone status, TC, and oxidative stress biomarkers (96). Marjoram herb significantly reduces INS and HOMA-IR levels. Marjoram improves insulin sensitivity and reduces adrenal androgen levels in PCOS women (97). A summary of this section is shown in **Table 3**, a summary of the effect of single herb or herbal extract on PCOS is shown in **Table 4**.

**Table 3:** Animal experiment of treating PCOS with TCM.

Model	Intervention	Secrete factors	Results	Reference
PCOS rats; granulosa cells (GC)	Kidney nourishing and phlegm removing	FBG, TC, TG, LDL, HDL, FFA, APN	The contents of FBG, TC, TG, LDL, and FFA in serum decreased, while the expression of HDL increased. The expression level of APN in serum, AdipoR2 mean density value in ovarian GC membrane, p38MAPK mRNA and FATP1 mean density value in GC cells increased	2018 (42)
PCOS rats	Removing stasis and phlegm method	T, LH, FSH	It can improve PCOS hyperandrogenemia, improve granulosa cell layer number and luteal tissue number, regulate LH/FSH ratio and glucose metabolism	2015 (44)
PCOS rats	Bushen Jieyu Tiaochong Formula	FT, LH, FSH	The abnormal follicular cystic dilation was improved, and the serum FT and LH levels and LH/FSH ratio were decreased	2021 (46)
Obese PCOS rats	Cangfu Daotan Decoction	TC, TG, LDL-C, LH, T, IL-1β, IL-6, TNF-α, HDL, FSH, E2	Serum TC, TG, LDL-C, LH, T, IL-1β, IL-6, and TNF-α levels were decreased, and HDL, FSH, and E2 levels were increased in a dose-dependent manner	2021 (76)
PCOS rats	Bushen Zhuyun Decoction	/	The expression of PI3K/AKT/mTOR pathway proteins in PCOS rats was upregulated, and the expression of apoptosis-related proteins was downregulated to repair ovarian lesions and improve cell apoptosis	2021 (77)
PCOS rats	Cangfudaotan Decoction	FPG, FNS, FSH, LH, P, T, TNF-α, IL-1β, IL-6, CRP	The levels of FPG, FNS, and HOMA-IR decreased, LH and T decreased, and TNF-α, IL-1β, IL-6, and CRP were inhibited	2020 (78)
PCOS rats	Xiao Yao San	E2, P, LH, NE	The levels of E2 and P in serum decreased, while the levels of LH increased. The formation of vesicles decreased, and apoptosis and autophagy of granulosa cells decreased. The levels of dopamine β-hydroxylase and c-Fos in locus coeruleus increased, and the levels of NE in serum and ovarian tissue decreased	2017 (79)
PCOS rats	Dodder Total Flavone	T, GnRH, LH, E2, FSH	Serum T, GnRH, LH levels, ovarian index, and LH/FSH ratio were significantly decreased, while E2 and FSH levels were significantly increased. It can reduce the expression of AR in the hypothalamus, pituitary gland, and ovary	2019 (85)
PCOS-IR rats	Quercetin	INS, IL-1β, IL-6, TNF-α	The levels of INS, IL-1β, IL-6, and TNF-α in blood were decreased significantly, and IR was decreased	2017 (90)
PCOS rats	Shouwu Jiangqi Decoction	FSH, LH, T	Improves insulin resistance and ovulation disorders	2016 (91)
PCOS-IR rats	Liuwei Dihuang Pills	FSH, E2, P, LH, T	Serum FSH, E2, and P levels increased significantly, while LH and T levels decreased. Ovarian polycystic changes were reduced, and follicular atresia was reduced	2020 (69)

PCOS, polycystic ovary syndrome; TCM, traditional Chinese medicine; CRP, C-reactive protein; FBG, fasting blood glucose; FFA, free fatty acid; FPG, fasting plasma glucose; FSH, follicle-stimulating hormone; FT, free testosterone; HDL, high-density lipoprotein; LDL, low-density lipoprotein; NE, norepinephrine; TC, total cholesterol; TG, triglyceride.

**Table 4:** Summary of a randomized study on the effect of single herb or herbal extract on PCOS outcomes.

Model	Herbalmedicine	Secrete factors	Results	Reference
RCT	Fenugreek Seed Extract	LH and FSH	The levels of LH and FSH were significantly increased, and the ovarian volume and ovarian cysts were significantly reduced	2015 (80)
RCT	Cinnamon bark	INS, TC, TG, LDL-C, HDL	Improved menstrual cycle and ovarian size	2019 (81–84)
RCT	Berberine	/	Reduce fat and improve insulin resistance	2016 (88)
/	Coptis chinensis	MAPK1, CXCL8, IL-6, IL-1β	The pathological damage of ovarian tissue was reversed and the mRNA and protein expression levels of central predictive targets (MAPK1, CXCL8, IL-6 and IL-1β) were improved	2021 (89)
RCT	Curcumin	INS, TC	Decreased body weight, TC, FBG, INS, IR, insulin sensitivity significantly increased	2020 (92–94)
RCT	S. officinalis extract	/	Reduced BMI and systolic blood pressure in PCOS patients and reduced insulin resistance	2020 (95)
RCT	Marjoram herb	FSH, LH, P, T, E_2, GnRH, DHEA-S, FPG	DHEA-s, fasting insulin and HOMA-IR levels were significantly decreased, insulin sensitivity was improved, and adrenal androgen levels were decreased	2016 (97)

LH, luteinizing hormone; FSH, follicle-stimulating hormone; INS, insulin; TC, total cholesterol; TG, triglyceride; LDL-C, low-density lipoprotein cholesterol; HDL, high-density lipoprotein; MAPK1, Mitogen-activated protein kinase; P, progesterone; T, testosterone; E_2, oestradiol; GnRH, DHEA-S, dehydroepiandrosterone-sulphate; FPG, fasting Plasma Glucose; HOMA-IR, homeostasis model assessment for insulin resistance.

### 2.4 Other

Qigong and Tai Chi are moderate-intensity exercises that can be traced back to ancient China. They can alter the autonomic nervous system, restore dynamic balance, reduce the stress associated with the HPOA, and regulate the balance of the autonomic nervous system to parasympathetic innervation. The effect of Qigong and Tai Chi on emotion regulation may be related to the regulation of the prefrontal cortex, limbic system, and striatum (98). It has been reported that Qigong and Tai Chi have a certain positive effect on the treatment of diseases. Qigong training can improve blood glucose status, and Tai Chi has a good effect on reducing blood glucose and glycosylated hemoglobin levels in patients with type 2 diabetes (99, 100). Tai Chi is better than brisk walking at reducing cardiovascular risk factors and improving psychosocial health (101). Baduanjin is a potentially effective choice for PCOS patients to improve their biomedical and psychosocial health (102).

Shao C et al. selected 80 PCOS patients with phlegm block as the study cohort and randomly divided them into a study group (RG) and a control group (CG). CG was treated with TCM decoction based on syndrome differentiation. RG was treated with Guijiaosan paste at Shenque point on the basis of CG treatment. Compared with the CG group, the BMI, endocrine function, IRI, and TCM syndrome scores were significantly improved in the RG group (p < 0.05) (36). Acupoint catgut embedding therapy combined with TCM is beneficial to improve blood glucose, blood lipid levels, and the pregnancy rate of obese PCOS patients with infertility (37). For patients with simple obesity secondary hyperlipidemia, Taijiquan combined with auricular point sticking treatment can show an obvious synergistic therapeutic effect (103). Combined treatment of acupuncture, moxibustion, and drugs can effectively improve endometrial receptivity and uterine artery blood flow in PCOS patients with kidney deficiency and blood stasis and increase pregnancy rate. The therapeutic effect of HOXA10 is better than that of Western medicine alone. Its mechanism may be related to the regulation of serum HOXA10 expression (104).

PCOS is the most common endocrine and metabolic disorder in women of childbearing age. TCM is based on the concept of holism, using multi-system regulation and multi-target action to treat PCOS. A large number of clinical practices have confirmed that TCM can effectively improve the symptoms and signs of patients, the ovarian microenvironment, the sympathetic nervous system, the endocrine system, and the disorder of glucose and lipid metabolism. There are various methods of TCM to treat this disease, from syndrome differentiation and classic prescription plus or minus treatment to single Chinese medicine, acupuncture, and moxibustion, etc.; the clinical efficacy is remarkable; the adverse reactions are small. In addition, the TCM formulation is flexible and can adjust the medication based on the basic prescription for different patients. The TCM formulation can comprehensively adjust the symptoms of patients in various directions and improve the psychological state of patients.

## 3 The mechanism of TCM in the treatment of PCOS

### 3.1 Affecting the menstrual cycle and ovulation function

PCOS is characterized by increased androgens and follicular stagnation, possibly due to an imbalance between AMH and FSH (20). EA has been reported to normalize FSH and AMH levels in granulosa cells by reducing overexpression of AMH, thereby improving follicular stasis in PCOS rats (105). Shi Y et al. used low-frequency EA at acupoints (CV-3 and CV-4) to treat the PCOS rat model. In the EA group, 80% of rats had recovered estrous cycle, improved ovarian morphology, reduced testosterone level, and increased peripheral blood E2 and P450arom levels. The expression of AMH and AMH type II receptors decreased, while the expression of FSH receptors increased (105). Animal experiments conducted by Xu G et al. showed that EA stimulation of CV4, SP6, and ST36 could reduce the number of immature follicles, LH/FSH, and serum AMH levels (106). A recent animal study showed that acupuncture improves ovulation disorder by downregulating LncMEG3 expression, inhibiting PI3K/AKT/mTOR pathway, and reducing granulosa autophagy (107). Another animal study showed that EA can upregulate the number of preovulatory follicles and corpus luteum by increasing the innervation of blood vessels (108).

Bushen Cupailuan Decoction can significantly improve the expression of aromatase mRNA and protein in GCs of PCOS model rats, which may be one of the mechanisms by which Bushen Cupailuan Decoction promotes follicular development and release (109). Baling Tiaogan Decoction can significantly improve clinical symptoms, increase endometrial thickness, reduce ovarian volume, and improve the clinical pregnancy rate. The mechanism may be related to the decrease of serum FFA and CRP levels and the increase of serum β-EP levels (59). The transcript levels of gonadotropin receptors (FSH receptor (FSHR) and LH), steroid receptors (Pgr and Esr1), and steroid proenzymes (Cyp19a1, Hsd3b1, Hsdl7a1, and Cyp11a1) are changed in the ovaries of PCOS rats. CHF ameliorates PCOS symptoms by improving the dysregulation of steroidal hormones and steroid enzymes in PCOS rats (110).

After the intervention of cryptotanshinone, the estrous cycle, body weight, ovarian coefficient, and ovarian morphology of PCOS model rats were improved, and the serum levels of T, androstenedione (A2), LH, and sex hormone-binding globulin (SHBG) were reversed. It may be related to the downregulation of CYP17 and AR gene expression (111). Dodder Total Flavone may regulate the secretion of estrogen and androgen and affect the HPOA to protect PCOS model rats (85).

### 3.2 Improving insulin sensitivity and relieving IR

PLA2G4A protein is essential in the pathogenesis of PCOS and diabetes. Acupuncture treatment can significantly downregulate the level of mir-32-3p, which regulates the expression of PLA2G4A protein (112). EA intervention can reduce PCOS-like symptoms in rats, and DHEA exposure-induced skeletal muscle autophagy deficits were improved. EA ameliorates IR, mitochondrial dysfunction, and endoplasmic reticulum stress by inactivating the mTOR/4E-BP1 signaling pathway (113). KNPR can effectively improve the glucose and lipid metabolism of obese female PCOS rats and increase the serum APN expression, the average density of AdipoR2 in ovarian GC membrane, and the average density of p38MAPK mRNA and FATP1 in GC cells. KNPK treatment of PCOS may be related to the activation of the APN/p38MAPK signaling pathway (42). The pharmacological effects of Zishen Yutai Pills (ZSYTP) on PCOS may be related to the HIF-1 signaling pathway, IR, and gene expression such as RNA splicing (114). ETAQC can upregulate the expression of p-AKT and GLUT4 in ovarian GCs and improve the degree of IR in ovarian tissue, thereby improving the quality of IVF-ET in PCOS patients (115).

PI3K/Akt signaling is one of the classical insulin signaling pathways; PCOS rats showed abnormal IR and PI3K/Akt signal transduction (116). Heqi San could restore serum hormone levels and ovarian morphological lesions and improve IR. Heqi San can change the expression levels of key factors of the PI3K/APT pathway, including p-ERK, p-AKT, p-GSK3β, IRS-1, PTEN, and GLTU4. Bioinformatics analysis showed that rno-miR144-3p, rno-miR-30c-2-3p, rno-miR-486, rno-miR-3586-3p, and rno-miR-146b-5p may play key roles in the occurrence of PCOS (70, 70). The study of Wang LH et al. showed that the mRNA expression and protein level of insulin receptor substrate 1 (IRS-1) and PI3Kp85α were significantly decreased in model group rats. Shouwu Jiangqi Decoction (SJD) can enhance the expression of IRS-1 and PI3K (91). Irs-1 Ser307 plays a key role in insulin signaling (117). LWDH Pills can significantly downregulate the phosphorylation level of IRS-1 (S307) in PCOS-IR rats and upregulate the phosphorylation levels of PI3Kp85 alpha, Akt, and FoxO1a and the mRNA levels of FSHR and Cyp19a1. IR can be improved by regulating PI3K/Akt signaling pathway (69).

Berberine could restore HOMA-IR and ISI values to normal levels and enhance GLUT4 expression. After berberine treatment, ovarian morphology also returned to normal. Zhang N et al. found that berberine may reduce the PCOS pathology and IR value through PI3K/AKT activation and MAPK pathway inhibition of GLUT4 upregulation (118). Berberine significantly decreased the expression of mTOR mRNA and increased the expression of IRS-1 mRNA in GCs. Berberine enhances insulin sensitivity by modulating the IRS-1 signaling pathway and mammalian target of rapamycin (mTOR) in patients with PCOS (119).

### 3.3 Oxidative stress

Sterol regulatory Element Binding protein-1 (SREBP1) expression increased in PCOS rats, and overexpression of SREBP1 inhibited the phosphorylation of insulin receptor β and AKT in primary GCs; these can aggravate the mitochondrial dysfunction and oxidative stress. EA treatment inhibits SREBP1 expression by inducing the activation of the AMP-activated protein kinase (AMPK) signaling pathway in PCOS-like rats. EA intervention ameliorates IR, mitochondrial dysfunction, and oxidative stress by regulating lipid metabolism regulator SREBP1, thus alleviating PCOS-like symptoms (120). Sirtuin 3 (SIRT3) expression was significantly decreased in GCs of PCOS patients (121). Bu-Shen-Tian-Jing Formula effectively alleviated the pathogenesis of PCOS by improving oxidative stress and glucose metabolism *via* mitochondrial SIRT3 and the insulin-induced PI3K/AKT signaling pathway (122).

Cangfu Daotan Decoction can reduce ROS protein expression in PCOS ovarian granulosa cells, correct oxidative stress *in vivo*, and improve the rate of high-quality embryos. Network pharmacologic analysis revealed the AGE-RAGE signaling pathway, endocrine resistance, IL-17 signaling pathway, prolactin signaling pathway, and hypoxia-inducible factor 1 (HIF-1) signaling pathway in diabetic complications. In addition, PI3K-Akt, IR, Toll-like receptors, MAPK, and AGE-RAGE were associated with PCOS (123). Erxian decoction (EXD) was used to treat PCOS. PI3K-Akt, insulin resistance, toll-like receptor, MAPK, and AGE-RAGE were associated with EXD treatment of PCOS (124).

### 3.4 Chronic inflammation

Chronic inflammation is accompanied by the occurrence and progression of PCOS. The expression levels of IL-6 and TNF-α in PCOS rats are significantly increased, and the Compound Malt Pill (CMP) treatment can reduce the expressions of IL-6 and TNF-α (125). Zhu Y et al. found that the Guizhi Fuling pill could improve the IR of PCOS patients by regulating intestinal flora and controlling inflammatory response (9, 9). TCM could inhibit the expression of serum LPS and TLR4, thus inhibiting the activation of NF-κB signaling-mediated inflammatory response in ovarian tissue (126).

Elevated levels of serum inflammatory cytokines IL-17a, IL-1Ra, and IL-6 lead to long-term subclinical inflammation in women. Abnormal changes in inflammatory factors can disrupt a woman’s normal ovulation and fertilization system, leading to PCOS, which is characterized by thinner periods and abnormal ovulation. TCM can effectively reduce oxidative stress-related complications in PCOS patients (92, 93). *C. chinensis* plays a therapeutic role in PCOS by regulating mRNA and protein expression levels of MAPK1, CXCL8, IL-6, and IL-1β (89). Quercetin significantly reduced the levels of insulin, IL-1β, IL-6, and TNF-α in blood and NF-κB nuclear translocation in GCs. This may be related to the inhibition of toll-like receptor/NF-κB signaling pathway and the improvement of the inflammatory microenvironment in ovarian tissue of PCOS rats (90).

### 3.5 Apoptosis

Wang C et al. found that CFD could improve IR, restore serum hormone levels, inhibit inflammatory cytokines, and reduce ovarian morphological damage and apoptosis in PCOS rats. It was related to the regulation of gene expression mediated by the IGF-1-PI3K/Akt-Bax/Bcl-2 pathway (78). BJTF may improve reproductive endocrine homeostasis and promote follicular development and ovulation by inhibiting the PERK-ATF4-CHOP signaling pathway. In addition, the expression of GRP78 was downregulated, which delayed the apoptosis of ovarian GCs mediated by ER stress. Moreover, BJTF can improve the behavioral performance of rats by regulating brain monoamine neurotransmitters (46). BSZYD can repair ovarian lesions and improve apoptosis through the ERα-mediated PI3K/AKT/mTOR pathway (77).

**Figure 1:**
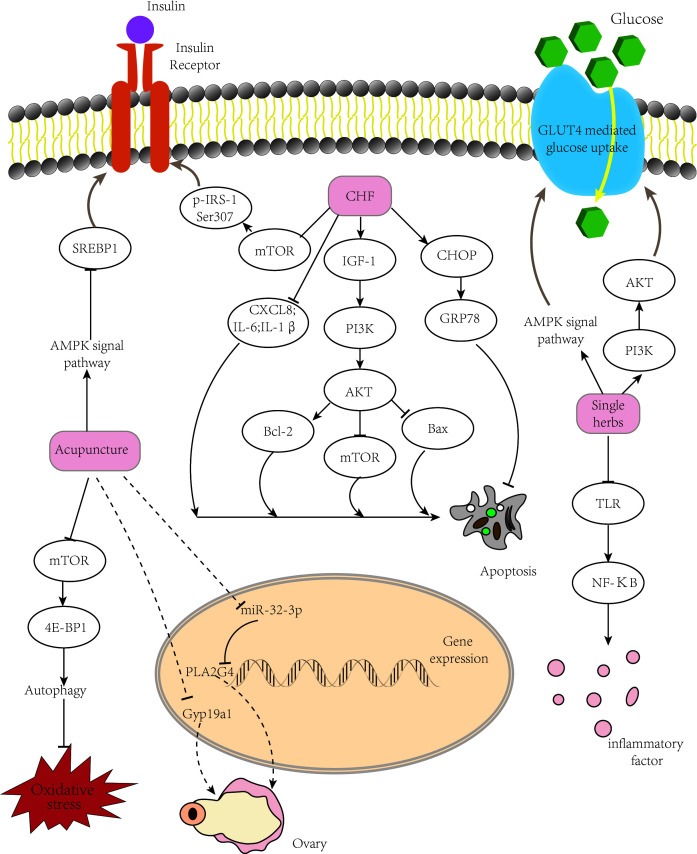
Mechanism of TCM treatment of PCOS. TCM ameliorates insulin resistance and improves follicular development *via* several pathways. TCM, traditional Chinese medicine; PCOS, polycystic ovary syndrome.

A summary of the mechanism is shown in **Figure 1**.

## 4 Discussion

PCOS is an endocrine syndrome with a relatively complex etiology and pathogenesis and various clinical manifestations. There are some disadvantages of Western medicine in the treatment of this disease, such as numerous side effects (127), and TCM is relatively safe in improving the symptoms of endocrine and metabolic disorders in PCOS patients. The advantage of TCM treatment of PCOS lies in syndrome differentiation, in-depth analysis of the primary and secondary relationship of pathogenic factors in different stages, grasping individual characteristics, and closely following the syndrome type to establish an individualized treatment plan and can achieve good clinical efficacy. TCM treatment of PCOS based on syndrome differentiation has made some progress in clinical research. This article describes the mechanism of TCM treatment of PCOS, including correcting endocrine hormone disorder, gene expression, and regulatory factors; improving IR; correcting lipid metabolism disorder; and improving pregnancy outcomes (128, 129).

TCM has been shown to be effective in treating PCOS. However, so far, the understanding of the etiology, pathogenesis, and treatment of TCM is not uniform, and TCM treatment still lacks evidence-based medical evidence. The proposed prescription drugs are mainly based on the clinical experience of doctors. A set of standard treatment plans has not been formed, and further research, exploration, and summary are needed. In addition, TCM focuses on overall efficacy and clinical safety but lacks accurate analysis and monitoring. At present, there are not enough studies on the pharmacodynamics and toxicology mechanism of TCM, and the active ingredients of the drug are unclear. Based on the advantages and disadvantages of TCM treatment of PCOS, new technologies and means can be adopted in the future to screen out the pharmacoactive components, identify the bioactive compounds, predict their related targets, and clarify the molecular mechanism of action and toxicological effects of Chinese herbal medicine in order to improve the curative effect of TCM treatment and make it the preferred treatment method.

## Author contributions

HC, CD, and ZM have contributed equally to this work and share first authorship. All authors contributed to the article and approved the submitted version.
